# Evaluation of Tail Lesions of Finishing Pigs at the Slaughterhouse: Associations With Herd-Level Observations

**DOI:** 10.3389/fvets.2021.650590

**Published:** 2021-07-15

**Authors:** Mari Heinonen, Elina Välimäki, Anne-Maija Laakkonen, Ina Toppari, Johannes Vugts, Emma Fàbrega, Anna Valros

**Affiliations:** ^1^Department of Production Animal Medicine and Research Centre for Animal Welfare, Faculty of Veterinary Medicine, University of Helsinki, Helsinki, Finland; ^2^Animal Sourcing, HKScan Finland Oy, Forssa, Finland; ^3^Animal Health ETT, Seinäjoki, Finland; ^4^Animal Welfare Program, Institut de Recerca i Tecnologia Agroalimentària, Monells, Spain

**Keywords:** tail biting, pig welfare, herd health, tail scoring, abattoir, harmful behavior

## Abstract

The prevalence of tail lesions evaluated at the slaughterhouse varies considerably between herds. These lesions result mainly from tail biting, a harmful behavior with multifactorial origin. This study sought to investigate if a batchwise inspection of tails at slaughterhouse could be a useful method to estimate the animal welfare situation in finishing pig herds, and if so, what type and detail of tail scoring such an inspection should utilize. We investigated the distribution of different types of tail lesions and how well their scoring at slaughterhouse was associated with the situation recorded on-farm by a veterinarian as part of routine herd health visits. We also wanted to determine if animal welfare-related herd-level parameters, recorded by herd veterinarians during herd health visits, are associated with tail scoring at the slaughterhouse. A total of 10,517 pigtails from 84 herds were scored for this study. Herd data were collected from the national health classification register for pig farms in Finland and also included annual herd production quality data collected by the slaughterhouse. The scores of the tails varied considerably between the herds. On average, 48.1% (sd = 19.3) of the tails with an average length of 30.4 cm (sd = 2.7) were fully intact, 37.3% (13.9) had healed (length = 26.4, sd = 5.1 cm), 12.4% (9.0) (length = 28.9, sd = 4.3 cm) had minor acute wounds, and 2.3% (2.1) (length = 24.2, sd = 6.0 cm) had major acute wounds. Proportions of different tail lesions at slaughterhouse were associated with or tended to be associated with the following herd-level parameters in regression models: use of wood as enrichment (*p* < 0.1), one health parameter (leg problems other than arthritis, *p* < 0.05), and long-term animal welfare estimate (annual mortality, *p* < 0.05). Detailed tail evaluation at the slaughterhouse shows potential in estimating the tail lesions and long-term welfare level on the farm. By recording only one type of tail condition (such as tails with major acute lesions) at the slaughterhouse, it is not possible to estimate the total tail lesion situation in the herds before slaughter. A more detailed scoring similar to the one used in this trial is recommended.

## Introduction

Tail biting (TB) is a major welfare concern in pig production (1) and also leads to large economic losses (2). Tail biting results in tail lesions (TL) and is related to several other problems, such as infection in the bitten pig (3), stress and pain in pigs (4, 5), carcass condemnations (6–9), and reduced daily gain (10). TB is a harmful behavior and has a multifactorial origin. Both suboptimal environmental, health and management-related factors, and certain characteristics of individual pigs are risk factors for TB (11). The magnitude of the number of possible risk factors is illustrated in the study of Taylor et al. (12), who collected altogether 83 risk factors for their husbandry advisory tool. In addition to being risk factors for TB, these parameters have an important effect on the welfare of pigs. Thus, tail lesions in the herds could be used as a simple measure of animal welfare, either at herd level before slaughter or at slaughterhouse during meat inspection. Previous studies have combined the estimation of tail lesions at the slaughterhouse and herd-level data (13, 14). Meat inspection of pigs has been suggested as a welfare surveillance tool (8), and other research has shown potential to use TL scoring as indicators of pig welfare on farms (15–18).

The prevalence of TL varies considerably in scientific studies and has been estimated to be 2–4 fold higher in pigs with undocked tails than those with docked tails (19). Sampling methods and definitions for TL differ and therefore studies often cannot be compared. The results differ especially when the tails were evaluated in the herd before slaughter or the data were collected from routine meat inspection data. In addition, it has been common in slaughterhouse studies to include mild lesions in the same score with healed lesions (7, 18, 20–24) or healed lesions have not been mentioned separately in the scoring definitions (9, 25). Only a few studies report healed tails separately from other tail lesions (6, 26). It is also not common to report the variability of different lesions in the slaughter batches. For example, the results from some studies show that severe tail lesions can be found in an average of 1.2–1.9% of the tails scored at the slaughterhouse; the minimum in all studies was 0% and the maximum varied between 7 and 22.9% (9, 18, 23). We consider both of these parameters very important. Healed tails can give an indication of when the tail damage occurred and knowledge on the variability of different TLs in the batches may help in preventing different types of TB in the herds (12).

Our aim was to investigate if a batchwise, thorough inspection of tails at the slaughterhouse could be a useful method to estimate the animal welfare situation in finishing pig herds, and if so, what type and detail of tail scoring such an inspection should utilize. To achieve this, we investigated (a) how the distribution of different types of intact and damaged tails vary between farms, (b) how well TL scoring at the slaughterhouse is associated with the herd-level situation of tails scored during a single herd health visit, and (c) how long term production parameters (annual mortality, annual total, and partial carcass condemnations) are associated with tail scores in the slaughterhouse. In addition, to further evaluate the value of TL scoring at the slaughterhouse as a proxy measure of on-farm conditions: (d) we tested if certain relevant, and available, risk factors for tail biting (mainly related to health and use of enrichment), recorded by herd veterinarians during herd health visits, are associated with tail scoring at the slaughterhouse. We hypothesized that by recording only the severely bitten pigs at the slaughterhouse we could estimate the total TL level in the herd, both in the long and short term. We also hypothesized that certain herd-level parameters, such as animal health and management features, are associated with the level of tail damage scored at the slaughterhouse.

## Materials and Methods

### Tail Scoring at the Slaughterhouse

The data collection from undocked finishing pigs has been described in detail by Valros et al. (26). Here we describe only the main points of scoring. The data were collected at a large Finnish slaughterhouse over 5 consecutive days in June 2019. Most of the carcass tails handled at the slaughterhouse were scored. In this study, we included only the tails from the herds that had sent at least 50 pigs to slaughter during the data collection period of 5 days, leaving a sample of 10,517 tail scores from 84 herds (see more in data handling and statistical analyses). We wanted to ensure better data quality by including only herds large enough to yield data not only from a few occasional animals but also from a large number of pigs. The carcasses were hanged on gambrels ensuring the identification of the farm or origin. Carcasses were identified by the herd tattoo and individual slaughter data was recorded per pig via the gambrel identification system. Pork producers selling their finishers to slaughter during that week did not know about the study. This dataset includes a subsample of the carcasses reported in another article (26), where the average carcass weight of the pigs was 89.7 kg (sd = 7.38 kg).

Pictures and live observations were used to train the researchers to harmonize the scorings before the actual data collection at the slaughterhouse line. The scoring point was situated after singeing, whipping, bung drilling, and chest opening. Altogether six researchers, two at a time, were responsible for the scorings, which took about 7–8 s per tail due to slaughterhouse line speed. One of the two main scorers was present during all scoring sessions. The scorers were standing on a platform high enough for them to palpate and measure each tail easily. One researcher measured the tail of the carcass with a 50 cm long ruler and assessed the possible tail lesion while the other recorded the observations. With the carcasses hanging upside down, the end of the ruler was placed on the dorsal side of the tail and firmly pushed toward the base of the tail. The tail was then manually extended against the ruler. During scoring, the observers consulted each other actively in case of questionable scorings.

The scoring system used included elements from the systems developed by The FareWellDock-consortium (27) and those suggested by the pig welfare subgroup of the EU animal welfare platform (28). The preliminary system was amended to be usable at the slaughterhouse line after the pilot visit testing. A tail with multiple lesions was scored according to the most severe lesion. Tails scored as healed were also checked for acute damage. Finally, the tails were classified as intact, healed, with minor acute wounds, and with major acute wounds (Table 1). Additional details are available in Valros et al. (26).

**Table 1:** Tail scoring system used at the slaughter line after scalding of the carcass and the mean percentage and standard deviation of one delivery group of finishing pigs from 84 herds (10,517 carcasses) according to their tail lesion scores and tail length in centimeters.

**Tail score**	**Definition**	**Mean (sd) percentage of tails**	**Tail length, mean, cm (sd)**
Intact	The tail is fully intact, the end is rounded, and slightly flattened	48.1% (19.3)	30.4 (2.7)
Healed	The tail is clearly shortened; the tail end is scarred, of abnormal shape or too thick to be intact. The skin is totally healed (no scab, wound, or missing tissue)	37.3% (13.9)	26.4 (5.1)
Minor acute wound	The tail has missing tissue, which has not yet fully healed; uneven “dents” in the skin; or a part of the tail is missing. Wound is >0 cm but <2 cm in diameter or length	12.4% (9.0)	28.9 (4.3)
Major acute wound	The tail has missing tissue, which has not yet fully healed; uneven “dents” in the skin; or a part of the tail is missing. Wound is ≥2 cm in diameter or length	2.3% (2.1)	24.2 (6.0)

### Data Collection From the Herds Through the National Herd Health Register, Sikava

Data concerning the finishing pigs in 84 herds were collected from the national health classification register for pig farms in Finland, Sikava (www.sikava.fi). The Sikava system has been ISO9001:2015 certified since 2014. The national level of Sikava fulfills the national food quality schemes criteria as described in Article 16 of Regulation (EC) No 1305/2013 and was recognized by Finnish Food Safety Authority EVIRA in 2013. This classification system covers ~90% of Finnish swine farms and over 95% of the pork production in the country. All herds of this study fulfilled the national level requirements of Sikava.

Detailed information of the health classification system can be found at www.sikava.fi. Here, we present only the most important features of the system relevant for this study. The herds at the national level of Sikava make a healthcare agreement with a veterinarian who has participated in a course organized by Sikava. The farm must invite the herd health veterinarian to visit the herd four to six times a year or at least once in every batch in all in-all out finishing units and the veterinarians need to document their observations in an internet-based database with pre-coded evaluations. The visit interval is controlled by Sikava officers and delayed visits affect animal trade. The farm must be at a national level of Sikava before slaughter of the pigs.

In this study, we included observations about pig health, behavior and use of enrichment for the pigs recorded by the veterinarian during the herd health visit according to the scoring system acquired by Sikava (Table 2). The results of a single herd health visit record from each herd were extracted from the Sikava database. We used the latest visit documented by the herd health veterinarian before the data collection at the slaughterhouse. To ensure the visit included observations on the pigs assessed for tail damage at the slaughterhouse, only herds that had been visited between >1 week and <3 months before the first sampling day were included. This time period was an average of 36.8 (sd = 21.8) days in our 84 study herds.

**Table 2:** Summary of the data collected by the herd health veterinarians in finishing units of 84 herds during routine Sikava herd health visits.

	**Scoring**			
**Evaluation of the pigs, scores**	**0**	**1**	**2**	**3**
1. Percentage of pigs with bitten tails	None or single pigs	Some, 1–5% of the pigs	Several, 6–10% of the pigs	Plenty, >10% of the pigs
2. Disease symptoms (arthritis, claw injury, leg problem other than arthritis, coughing, sneezing, diarrhea, skin injury or infection, abscesses, central nervous system symptoms, runts, and hernia)	None or single pigs	Some, 1–5% of the pigs	Several, 6–10% of the pigs	Plenty, >10% of the pigs
3. Evaluation of the percentage of intact tails (=a tail with full length and curled up. If a tail has signs of healed or acute TB and the tail is shortened/damaged/stuck between legs, it is not intact).	Intact >95% of the pigs	Intact >80% of the pigs	Intact >70% of the pigs	Intact <70% of the pigs
**Behavioral measures, scores**		1 = Good	2 = Satisfactory	3 = Poor
1. Explorative behavior to given material. Behavior of standing animals if they are not eating, drinking defecating or urinating. The behavior should be directed toward enrichment material, not toward pen structures or pen mates.		>70% of pigs explore enrichment	40–70% of pigs explore enrichment	<40% of pigs explore enrichment
2. Reaction of the pigs to the examiner, evaluated after examiner was first walking from one end of the corridor to another. The examiner did not enter the pen.		Pigs approach the examiner within few minutes	Only some pigs dare to approach the examiner	Pigs do not approach the examiner, are afraid, stay in the back of the pen
**Environmental measure**				
Use of materials as environmental enrichment, the material used was recorded (Toy, straw, sawdust, wood, paper, peat, hay, wood chips, other)	0 = Not used	1 = Yes, used		
**Collection of data from herd records**				
Inter-visit mortality	% of finishing pigs dead or euthanized after the previous visit			

The slaughterhouse continuously collects the following information on parameters for follow up of production quality and saves this data in Sikava database: annual mortality, annual percentage of carcasses with total carcass condemnation, and annual percentage of carcasses with partial carcass condemnation. The herd owners and health care veterinarians can see these parameters and use them as long-term animal health and welfare estimates for the farms and follow-up of production quality. In this project, we refer them as production parameters. They were available for 79 herds of this study for the period from July 2018 to June 2019.

### Data Handling and Statistical Analyses

All statistical analyses were performed using IBM SPSS statistics version 25. The unit of interest was the herd.

At first, the data were checked (26) and yielded a data set of 14,382 tails scored out of a total of 14,433 original scorings. After including only the herds that had sent at least 50 pigs to slaughter during the study week and from which the herd health visit data were available (84 herds), altogether 10,517 tails (mean of 215.2 [sd = 83.0] tails per herd) were included in the dataset of this study.

Continuous variables were assessed for normality visually and using the Shapiro–Wilk test. The correlation between the percentages of different tail scorings at the slaughterhouse was calculated with Pearson correlation. Differences between farms within different tail lesion percentage class, based on data collected by the veterinarian during the heard health visit (tail lesions below 1 vs. 1–10%) in the tail scoring at the slaughterhouse was evaluated using separate *t*-tests for each tail lesion outcome. Similarly, the difference between farms within the different percentage of intact tails-classes based on the herd health visit (≤80%, >80%, and >95% intact tails) in their percentage of tail outcomes at the slaughterhouse was tested using one-way ANOVA tests, followed by Bonferroni-correction for pairwise tests. After calculation of the descriptive data preliminary univariable statistical analyses were performed to find the associations between the predictor variables (collected from Sikava database, see Table 2) and the four different outcomes one at a time (percentage of tail scores: intact, healed, minor acute wounds, and major acute wounds). Two new variables were formed by combining the evaluations performed by the veterinarians during the herd health visits. Firstly, “skin problems” represented the sum of the scores of the variables “skin injuries/infections” and “abscesses.” Secondly, a total symptom score was calculated as the sum of the scores given to all 11 symptoms (scored each as 0, 1, or 2 and thus yielding a maximum total score between 0 and 22). This score was used as an estimate of the overall health situation on the farm. Univariate associations between predictor variables and outcome were evaluated using *t*-test (two possible values) or ANOVA (more than two possible values of the predictor variable). The correlations of the continuous predictor variables (inter-visit mortality, farm size, and total symptom score) and the four different outcomes were calculated separately with Spearman rank correlation (non-normally distributed data). A liberal *p*-value of 0.1 was used as a keep-in or drop-out threshold for biologically relevant variables to be included in the regression models. Associations between predictor values left in the models were assessed and determined as non-significant.

Finally, based on the univariate test results four different multivariable linear regression models were built for the different outcomes using a manual backward elimination model building strategy. Variables were excluded one by one based on non-significant *p*-values (>0.1) and the AIC-value. Originally, the model for the first outcome “percentage of intact tails” contained the following predictor variables: number of finishers in the herd, wood as enrichment (no/yes), leg problems other than arthritis (no/yes), inter-visit mortality, skin problems (no/yes), runts (no/yes), and total symptom score (0–24). Similarly, in the case of the second outcome “percentage of healed tails,” the following predictor variables were tested: number of finishers in the herd, inter-visit mortality, wood as enrichment (no/yes), leg problems other than arthritis (no/yes), skin problems (no/yes), runts (no/yes), and total symptom score (0–24). For the third outcome “percentage of minor wounds,” the following variables were tested in the model: number of finishers in the herd, inter-visit mortality, enrichment (good/satisfactory/poor), abscesses (no/yes), and runts (no/yes). In the fourth model building, the predictor variables number of finishers in the herd, wood as enrichment (no/yes), and explorative behavior (Score 1 or 2) were included in the model of the outcome “percentage of tails with major wounds.” *P*-values from pairwise comparisons of interaction terms were Bonferroni-corrected. The residuals of the final models were tested for their normality.

Correlations of the production parameters (annual mortality, annual total, and partial carcass condemnations) and the four different tail outcomes in the slaughterhouse (intact tails, healed tails, tails with minor wounds, and tails with major wounds) were tested separately with Pearson correlations (total and partial carcass condemnations) and Spearman rank correlation (annual mortality, which was non-normally distributed).

## Results

### Descriptive Statistics of Tail Scoring at the Slaughterhouse

Out of the 84 herds in this study, 69 (82.1%) were finishing units growing feeder pigs from a weight of ~30 kg until slaughter and 15 (17.9%) were farrow-to-finish herds. The production type did not affect the prevalence of the different tail scorings at the slaughterhouse (*p* > 0.05 for all). The study herds had room for an average of 1,407.0 (sd = 1,460.8) finishing pigs, and the average inter-visit mortality was 1.3% (sd = 0.9).

**Figure 1:**
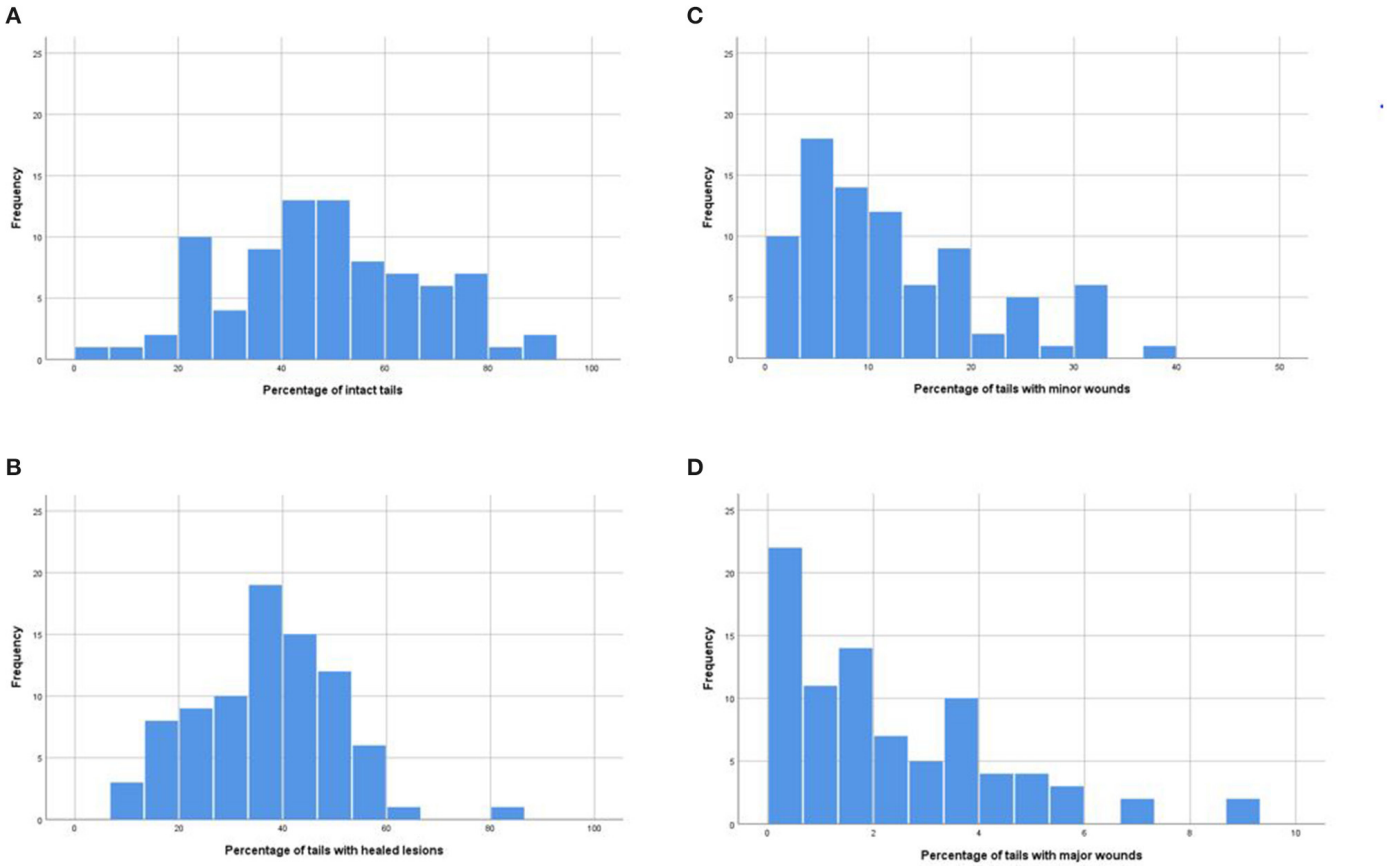
Frequency distributions of percentages of tail classifications in 84 herds: **(A)** intact tails, **(B)** healed tails, **(C)** tails with minor wounds, **(D)** tails with major wounds. Undocked tails of finishing pigs (*n* = 10,517) were evaluated at the slaughterhouse after scalding.

**Figure 2:**
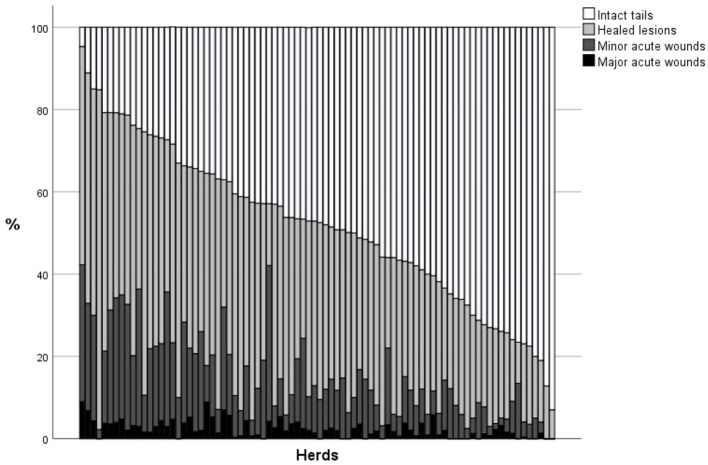
Study herds (*n* = 84) organized according to classification of the undocked tails of their finishing pigs in slaughterhouse: percentage of intact tails, tails with healed lesions, tails with minor acute wounds, and tails with major acute wounds ordered according to increasing proportion of intact tails.

On average, the mean percentage of fully intact tails at the slaughterhouse was 48.1% (see Table 1 for the tail classification results). The percentage of intact tails correlated negatively with the percentage of healed tails and tails with minor and major wounds (see Table 3 for all correlations between different tail classifications). Figure 1 shows the frequency distributions of tail classifications in the herds. The study herds had varying combinations of the percentages of tail findings (Figure 2).

**Table 3:** Correlation of percentages of herd-level tail evaluations from 84 Finnish herds.

**Percentage of tails**	**Healed**	**With minor wounds**	**With major wounds**
Intact	−0.858^**^	−0.706^**^	−0.539^**^
Healed		0.258^*^	0.270^*^
With minor wounds			0.522^**^

*Pearson correlation*,

** *p < 0.01 and*

* *p < 0.05*.

*Undocked tails of finishing pigs (n = 10,517) were evaluated at the slaughterhouse after scalding*.

### Descriptive Statistics of Sikava Herd Health Visit Recordings and Their Association With Slaughterhouse Tail Scoring

**Figure 3:**
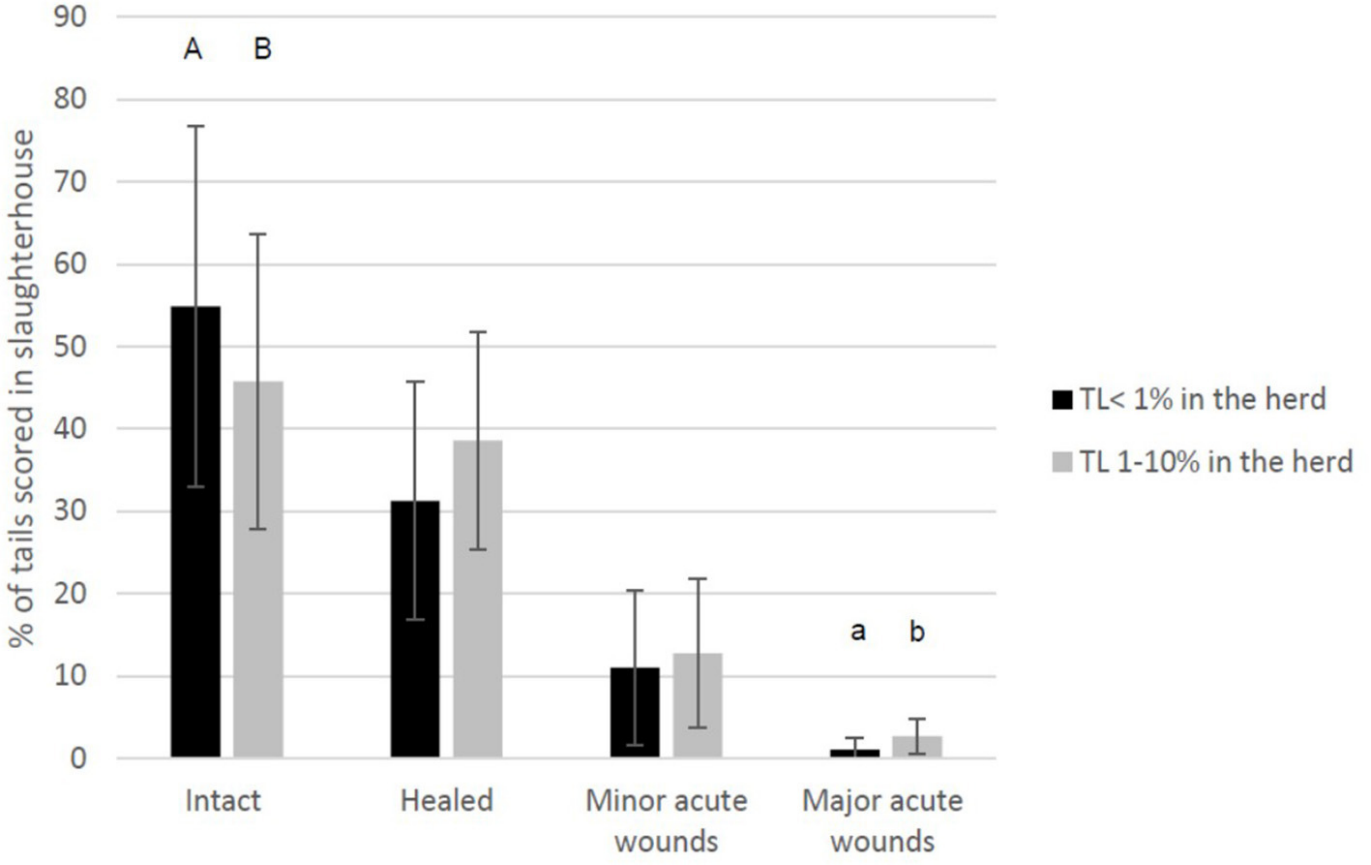
The average percentage of undocked tails of finishing pigs per herd (*n* = 84) scored at the slaughterhouse in four categories (intact, healed, with minor acute wounds, and with major acute wounds) and divided according to the evaluation recorded in the same herds during the herd health visit (TL, Tail Lesion <1%: no or single cases of tail-bitten pigs during the herd health visit, TL 1–10% of pigs with bitten tail). Within tail score comparisons, different letters represent statistical significance or tendency: ab *p* < 0.01, AB *p* < 0.1.

During the one herd health visit, the herd veterinarians recorded TLs in 21 (25%) herds in none or single animals. In addition, 58 (69%), 5 (6.0%), and 0 herds were given a score of 1 (1–5% of the pigs), 2 (6–10% of the pigs), or 3 (>10% of the pigs), respectively. Later in the analyses, the herds were divided in the following two groups: 21 herds with no or single animals with bitten tails and 63 herds with TL in 1–10% of the pigs. Figure 3 shows the different tail scores at slaughterhouse divided according to the evaluation of the herd health veterinarian during the herd visit. In the herds where the veterinarian had estimated the percentage of pigs with bitten tails to be <1% (score 0 = no or single animals with tail lesions) compared to the figure of 1–10% (Score 1 or 2, some or several), a statistically higher percentage of the tails were evaluated at the slaughterhouse as intact and a lower percentage as tails with major acute wounds.

**Figure 4:**
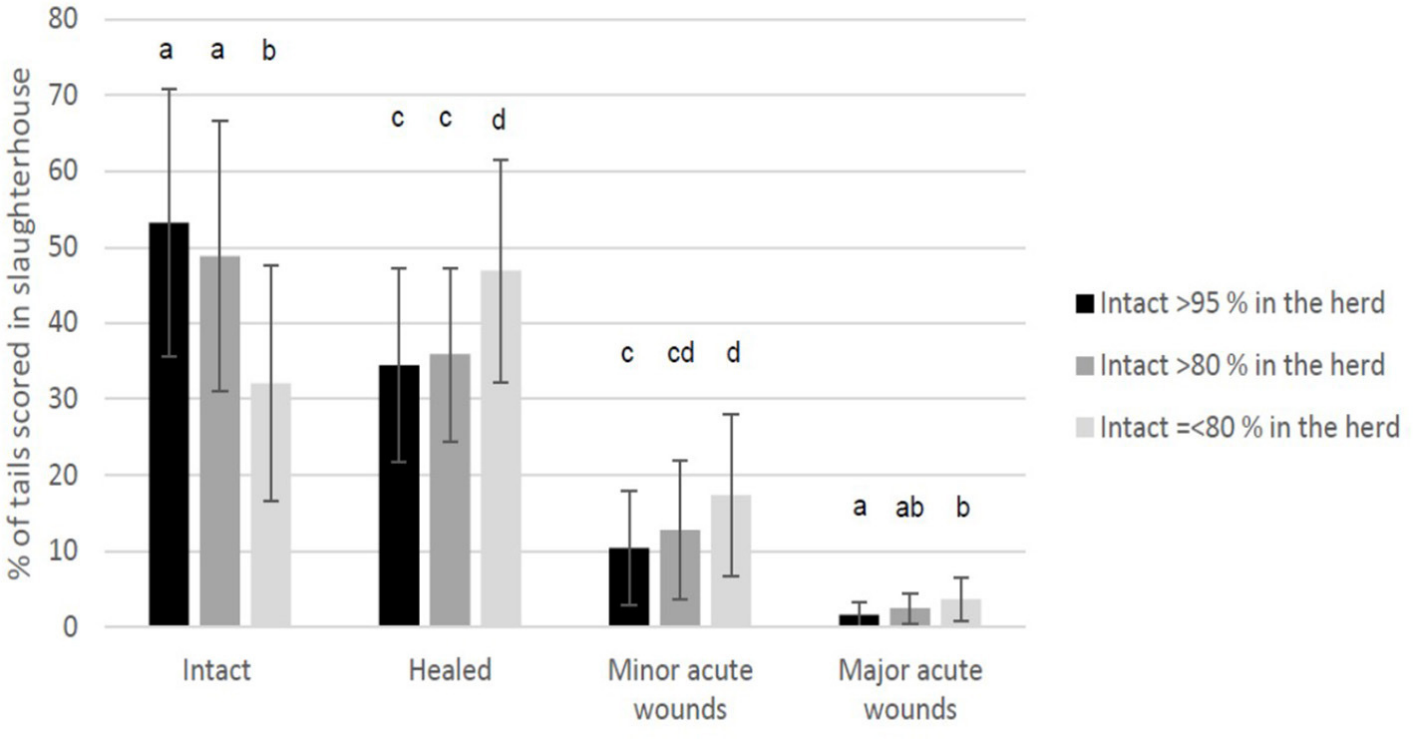
The average percentage of undocked tails of finishing pigs per herd (*n* = 84) scored at the slaughterhouse in four categories (intact, healed, with minor acute wounds, and with major acute wounds) and divided according to the evaluation of the percentage of intact tails recorded during the herd health visits (>95%, >80%, or ≤80%). Within tail score comparisons, different letters represent statistical significance: ab *p* < 0.01, cd *p* < 0.05.

The veterinarians scored the percentage of intact tails in finishers during the herd health visit as follows: in 43 herds (51.2%) > 95% of the tails were intact, in 23 herds (27.4%) > 80% of the tails were intact, in 16 herds (19.0%) > 70% of the tails were intact, and one herd (1.2%) had ≤ 70% intact tails. Figure 4 shows the different tail scores at slaughterhouse divided according to the evaluation of the percentage of intact tails by the herd health veterinarian during the herd health visit: when the herd veterinarian had evaluated the percentage of intact tails to be >95% in the herd compared to the figure of <80%, a higher percentage of the tails were scored at the slaughterhouse as intact and a lower percentage as tails with healed, minor acute or major acute wounds.

Descriptive herd data (84 herds) that were used in statistical analyses are shown in Table 4 (enrichment materials used) and Table 5 (disease symptoms evaluated by the veterinarian). The herd veterinarian had scored the explorative behavior to given material to be Good in 46 (54.8%), Satisfactory in 26 (30.9%), and Poor in 11 herds (13.1%). Similarly, the reaction to the examiner was scored to be Good, Satisfactory, and Poor in 79 (94.9%), 4 (4.8%), and 0 herds, respectively.

**Table 4:** Information recorded by veterinarian during one herd health visit on the use of different materials used as environmental enrichment in 84 study herds growing finishing pigs.

Material^*^	**0 = No**	**1 = Yes**
Toy	16 (19.0%)	68 (81.0%)
Straw	21 (25.0%)	63 (75.0%)
Sawdust	27 (32.1%)	57 (67.8%)
Wood	56 (66.7%)	28 (33.3%)
Paper	58 (69.0%)	26 (31.0%)
Peat	64 (76.1%)	20 (23.8%)
Hay	65 (77.4%)	19 (22.6%)
Wood chips	83 (98.8%)	1 (1.2%)
Other	82 (97.6%)	2 (2.4%)

* *The farms may have used more than one material*.

**Table 5:** The presence of different symptoms in the finishing pigs (number and percentage of herds out of 84) recorded by herd veterinarians during one herd health visit.

**Symptom**	**0 = None or single animals**	**1 = Some, 1–5% of the pigs**	**2 = Several, 6–10% of the pigs**	**3 = Plenty, >10% of the pigs**
Arthritis	10 (11.9%)	68 (81.0%)	6 (7.1%)	0
Claw injury	68 (81.0%)	16 (19.0%)	0	0
Leg problem other than arthritis	40 (47.6%)	44 (52.4%)	0	0
Coughing	73 (86.9%)	8 (9.5%)	3 (3.6%)	0
Sneezing	77 (91.7%)	7 (8.3%)	0	0
Diarrhea	66 (78.6%)	18 (21.4%)	0	0
Skin injury or infection	44 (52.4%)	40 (47.6%)	0	0
Abscesses	50 (59.5%)	34 (40.5%)	0	0
Central nervous system symptoms	79 (94.0%)	5 (6.0%)	0	0
Runts	36 (42.8%)	48 (57.1%)	0	0
Hernia	10 (11.9%)	71 (84.5%)	3 (3.6%)	0

### Regression Models for Outcomes Intact Tails, Healed Tails, Tails With Minor Wounds, and Tails With Major Wounds

**Figure 5:**
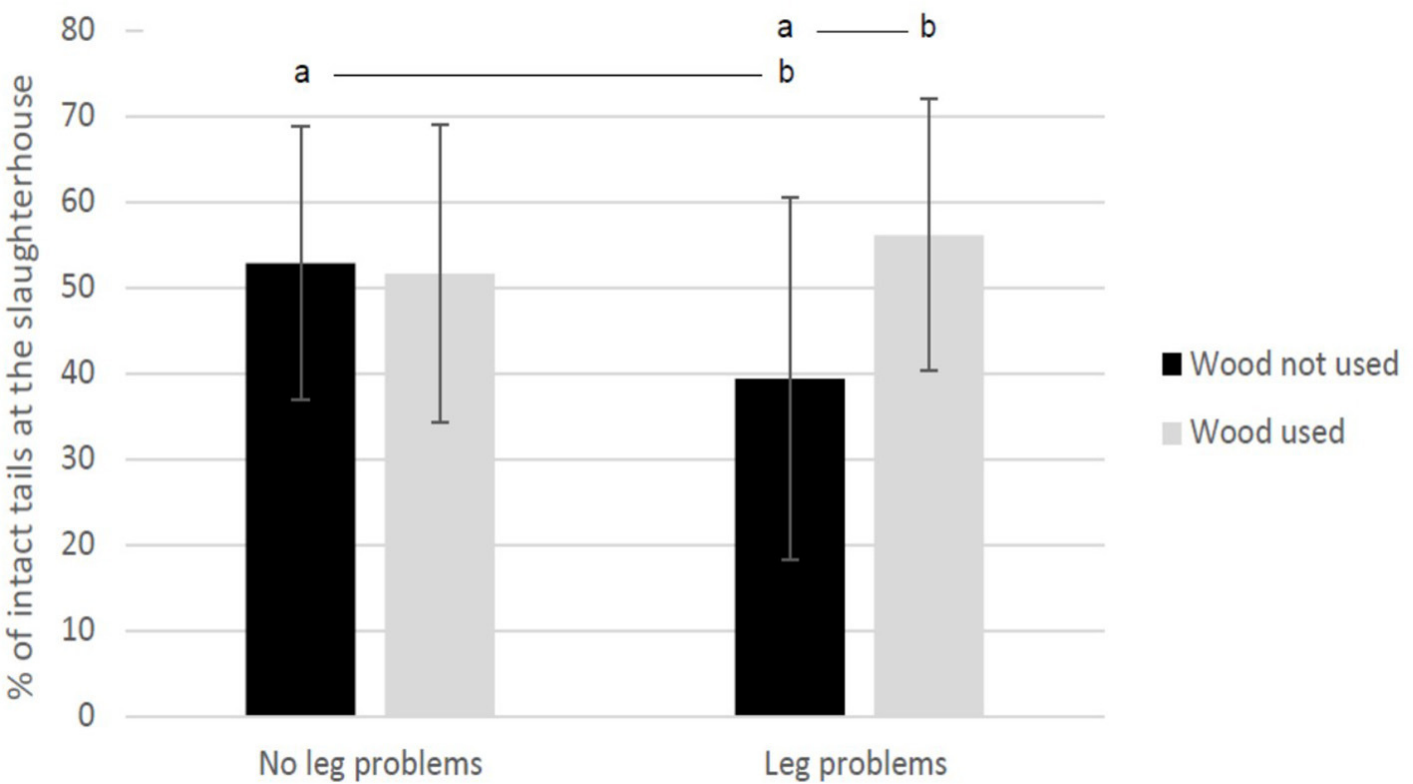
Percentage of undocked, intact tails of finishing pigs evaluated at the slaughterhouse. The pigs originated from 84 herds, where the herd veterinarian collected the data during one herd health visit. The results are shown separately for herds using wood as enrichment and having leg problems other than arthritis present in the herd during the herd visit (interaction in the regression model). Different letters represent statistical difference: ab *p* < 0.01.

The final regression model for percentage of intact tails (*R*^2^ = 0.175) included the following parameters: wood as enrichment (*df* = 1, *F* = 3.278, and *p* = 0.07), leg problems other than arthritis (*df* = 1, *F* = 1.552, and *p* = 0.2), inter-visit mortality (negative association: β: −4.4, *df* = 1, *F* = 4.270, and *p* = 0.04), and the interaction “wood as enrichment × leg problems other than arthritis” (*df* = 1, *F* = 3.931, and *p* = 0.05). In those herds, where the veterinarian had recorded leg problems other than arthritis, the percentage of intact tails was higher if wood was used as enrichment compared with the situation when wood was not used (Figure 5).

The final regression model for healed tails (*R*^2^ = 0.061) included one parameter “leg problems other than arthritis” (*df* = 1, *F* = 5.293, and *p* = 0.02). The herds with leg problems other than arthritis had an average of 40.5% (sd = 2.0) healed tails compared with no such symptom recorded (33.7%, sd = 2.1). Similarly, the regression model for percentage of tails with minor wounds (*R*^2^ = 0.045) contained one parameter, inter-visit mortality (β = 2.0, *df* = 1, *F* = 3.850, and *p* = 0.05), with the association being positive.

**Figure 6:**
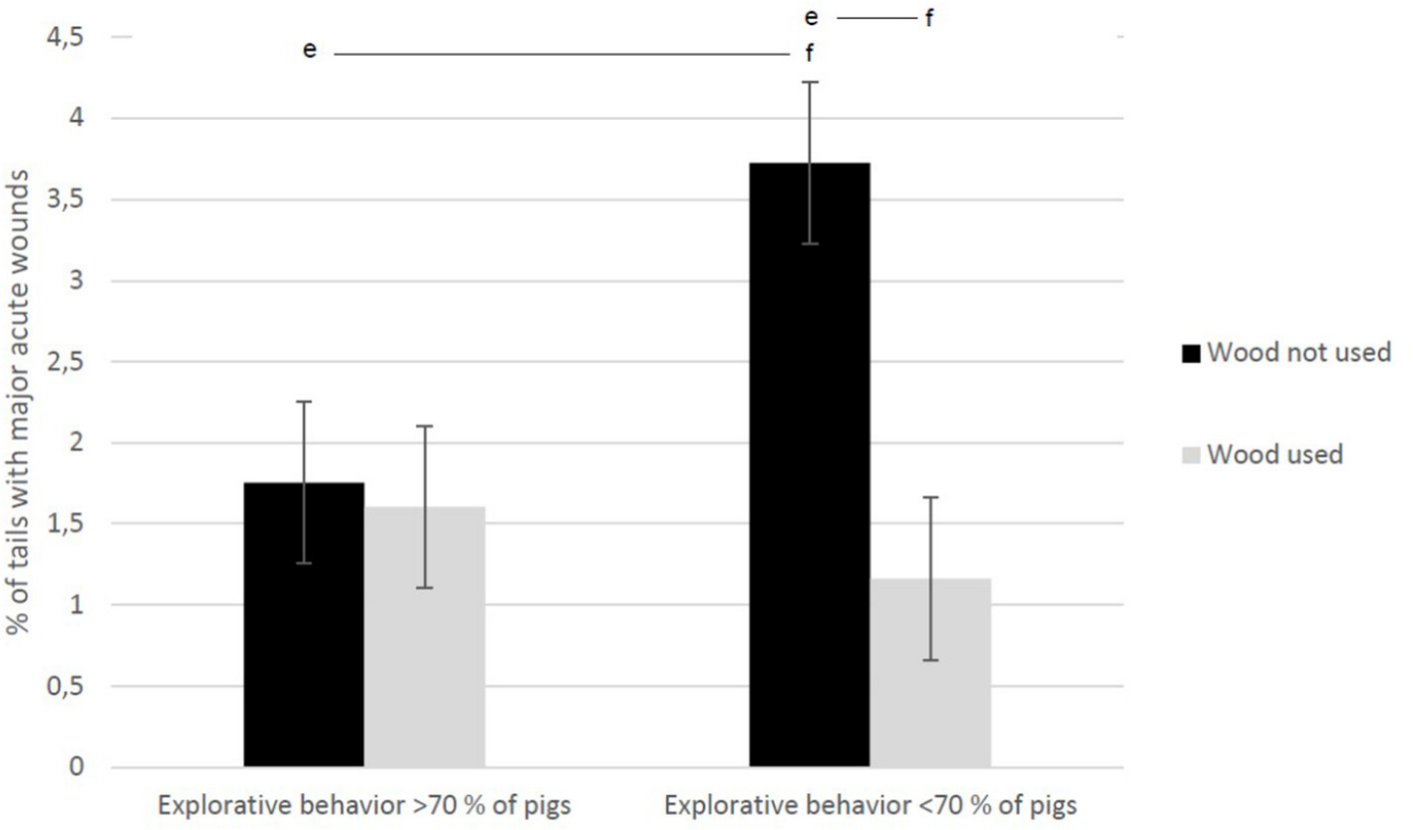
Percentage of undocked, tails with major acute wounds evaluated at slaughterhouse. The finishing pigs originated from 84 herds, where the herd veterinarian collected the data during one herd health visit. The results are shown separately for herds using wood as enrichment and according to explorative behavior of standing animals if they were not eating, drinking, defecating, or urinating (interaction), as evaluated by a veterinarian during the herd health visit. The behavior was directed toward enrichment material and not toward pen structures or pen mates. Different letters represent statistical difference: ef *p* < 0.001.

The final regression model for percentage of tails with major acute wounds (*R*^2^ = 0.238) included the following parameters: wood as enrichment (*df* = 1, *F* = 8.939, and *p* = 0.04), explorative behavior (*df* = 1, *F* = 2.845, and *p* = 0.1), and the interaction “Wood as enrichment × Explorative behavior” (*df* = 1, *F* = 7.086, and *p* = 0.009). Using of wood as enrichment was associated with fewer tails with major wounds, especially in herds where the pigs were recorded to show explorative behavior toward enrichment material (Figure 6).

### Production Parameters and Their Association With Slaughterhouse Tail Scoring

The production parameters (long-term animal health and welfare estimates of the herds) revealed an average annual mortality of 1.9% (sd = 1.88), an annual total carcass condemnation of 0.27% (0.17), and an annual partial carcass condemnation of 6.0%.

The percentage of intact tails in the slaughterhouse data tended to correlate negatively with annual total carcass condemnations (*r*_*p*_ = −0.21, *p* = 0.06), annual partial carcass condemnations (*r*_*p*_ = −0.21, *p* = 0.07), and annual mortality (*r*_*s*_ = −0.19, *p* = 0.09) of the herd. The percentage of healed tails in the slaughterhouse data correlated positively with annual mortality (*r*_*s*_ = 0.26, *p* = 0.02) but not with the measures of condemnations. The percentage of tails with minor wounds in slaughterhouse correlated positively both with annual total carcass condemnations (*r*_*p*_ = 0.30, *p* = 0.007) and with annual partial carcass condemnations (*r*_*p*_ = 0.26, *p* = 0.02) but not with annual mortality. The percentage of tails with major wounds scored in the slaughterhouse did not correlate with any of the parameters of production parameters (*p* > 0.1 for all).

## Discussion

Our study revealed that the prevalence of four different tail scores (intact, healed, minor wounds, and major wounds) in the herds varied considerably and herds commonly had different combinations of tail scorings evaluated at the slaughterhouse. Further, the correlations between the occurrence of healed and acute tail lesions were rather weak. Therefore, against our hypothesis, it is not possible to directly estimate the TL score at the slaughterhouse by recording only one type of pigtail score (such as major acute wounds of the tail at the slaughterhouse). As recorded by a veterinarian during one herd health visit, the tail score recorded at the slaughterhouse was only moderately related to the on-farm TB level. We further observed some herd-level factors and long-term indicators of herd health (one parameter, leg problems other than arthritis) and welfare status (production quality) that were associated with TL scores at the slaughterhouse, indicating that slaughterhouse tail scoring might be indicative about the overall welfare status of the farm. In particular, the tendency of the association between the using of wood as enrichment and tail scorings is interesting.

The fact that there was a great variation in the tail lesion profile (i.e., the distribution of different types of damage within different herds, Figure 2) supports the notion that there are different types of TB (29). This also indicates that TB is likely to occur at different stages of production in different farms. Some herds had a high percentage of healed tails, with almost no acute wounds. This indicates that TB is a problem at an earlier stage of production, possibly in the weaning unit. Other herds had a high percentage of minor acute wounds, but almost no major acute wounds. This might indicate a two-stage type of TB (29) or that the farm managed to intervene before TB became very severe. Further, some herds had a high percentage of major acute wounds, while only a moderate level of milder types of damage. This might indicate that the farms had suffered from sudden-forceful or epidemic-type TB (29, 30). Herds with a high percentage of all types of lesions are the most problematic; this indicates problems throughout the production period and a need to improve intervention and preventive strategies when outbreaks do occur. We suggest that by examining farm-wise lesion profiles it is possible to tailor advisory measures better for individual farms.

The scoring at the slaughterhouse was performed by the researchers and was much more detailed than the one performed by veterinarians in the herds. However, as shown in Figures 3, 4, farm evaluations on tail lesions and intact tails was somewhat related to the slaughterhouse evaluation of the tails. It is very difficult to evaluate tail damage clinically in live pigs (31) since they are in groups in their pens. Dark tails of some breeds may be more difficult to evaluate on farm and especially tails in large farms might be more difficult to evaluate. At the herd level, it is not possible for evaluators to check each tail separately by manual palpation, which would be needed for a detailed classification, especially to identify healed damages (26). One possibility is to examine tail posture in addition to lesions. In a study investigating tail position, finishing pigs with wounds or inflamed wounds were 4.15 and 14.24 times more likely to have hanging tails, respectively, than pigs with non-damaged tails (27). However, studies on tail posture in relation to tail lesions (27, 32) did not include scoring of healed tails. Thus, we do not know if tail posture is a relevant measure of healed tail lesions. In our data, it is likely that veterinarians included both intact and healed tails in the same category while evaluating the tail condition in the herd, especially if the healed tails were not drastically shortened and therefore the pigs were likely to keep them up and curled. The average length of the healed tails in slaughterhouse in this study was on average ~87% of the length of the intact tails. Valros et al. (26) suggested that healed tails with >75% of the average intact length remaining could be considered intact enough.

Healed lesions are problematic from both an animal welfare and economical perspective. A healed tail has been bitten at some point and caused pain and stress in the pigs. No information is available about the time needed for the tail to heal after being bitten. The wound caused by tail docking heals in 4 to 8 weeks (33). After biting, the time needed for healing might be even longer as there is strong inflammation after TB (3). Healed tails might still have macroscopically invisible deep infections (31) and their presence is associated with meat inspection findings and carcass condemnations at slaughterhouse (26). During the healing process, the tails are also likely to be promoting more TB than if the tails were totally intact. Furthermore, the timing of biting is difficult to evaluate after the tail has healed. Our slaughterhouse data show that the scores of both intact and healed tails are normally distributed. They are likely to describe the long-term situation both in the finishing herd and during earlier stages of the pig's life.

Meat inspection data could be better used in herd health and welfare planning at the farm level (8, 34). Our results suggest that tail condition at the end of the finisher phase and long-term parameters of production quality (total and partial carcass condemnations and annual mortality) provide similar information about overall welfare of animals in the herd. It has previously been shown that tail lesions have potential to be used as iceberg indicators of pig health and welfare (35). However, the scoring system probably affects the reliability of tail lesions as a measure of welfare. In the study by van Staaveren et al. (35), tails were scored with a five-level scale, which is not performed as routine at the moment of slaughter. Keeling et al. (25) also suggested that a three-level scale, including both old and new damage, should be used at slaughterhouse evaluations of the tails. However, based on our results, we carefully suggest that by evaluating the percentage of intact tails at the slaughterhouse it is possible to obtain a reasonable estimate of the long-term welfare situation on the farm.

Nearly half of the tails at the slaughterhouse were scored as intact, which is a very low figure. However, after dividing the tails into those without wounds (intact 48.1% and healed 37.3%, altogether ~85%) and those with wounds (minor 12.4% and major acute wounds 2.3%, altogether ~15%), the results seem to be in the same range with the results from other studies (21, 23, 29). However, comparison with the past studies is challenging as the data were collected from undocked or docked pigs and different scoring methods have been used. In addition, it is common to report the percentage of bitten tails and not the percentage of intact tails, which affects interpretation of the results. Finally, the definition of an intact tail in this study is very strict, and as suggested by Valros et al. (26), this level of precision is not feasible in a practical situation, such as where slaughterhouses would score tails on a continuous basis.

We showed that the results of different tail lesions at the slaughterhouse are associated with some herd-level parameters, such as use of enrichment. EFSA (11) recommends that pigs should be given suitable materials to enable them to fulfill their needs to look for food, bite, root, and manipulate. The material should be safe and at least one of the following qualities should be met: it should be edible/feed-like, chewable, investigable, or manipulable. All our study farms provided their pigs with enrichment of some kind. Most farms provided hay, straw, or both, which are known to reduce the risk of TB on farms (36). However, the data did not allow for evaluation of the actual functionality of the enrichment. Although a recent review concluded that enrichment other than straw was not effective in TB prevention (37), the finding in the current study of the effect (tendency) of the using of wood was consistent with the study by Telkänranta et al. (38). They found that provision of fresh wood in addition to a straw rack, a metal chain, and a daily provision of a small amount of sawdust to undocked finishers increased exploratory behavior while reducing tail and ear biting when compared with pigs given no wood. In our study, wood as enrichment tended to be associated with both the percentage of intact tails and tails with major wounds. Notably, wood was especially associated with an increase in the percentage of intact tails in herds with leg problems other than arthritis. Further, wood appeared to have a protective role against major tail wounds on farms where explorative behavior was assessed to occur at a low level. This supports the finding by Telkänranta and Valros (39) that providing freshly harvested (but not dry) wood had an additive effect with straw in reducing pen-mate manipulation. Possibly, there is something in the using of wood directing the innate need of the pigs to manipulate wood and not pen mates in some herds, especially when pigs face other challenges, such as leg problems. However, we did not analyze the combinations of different enrichments or their amounts used, and therefore this finding should be interpreted with caution.

Sickness has been considered as a risk factor for TB (12, 40). Two possible links between sickness and TB have been suggested (40). Recovered animals may have an increased propensity to become tail biters after illness or the increased attention of other pigs toward a sick one leads to an increased risk for the sick pig to become a victim of TB. The biggest health problems seemed to affect single individuals (usually in <5% of the pigs) of this study, such as different kinds of leg problems (arthritis, claw injuries, and other leg problems), presence of runt pigs or pigs with hernias, and skin injuries, infections, or abscesses, most of them likely to be associated with managemental problems. In univariate analyses, some of these conditions were associated with the tail scores, but in the final regression analysis only the presence of leg problems other than arthritis were associated with the percentage of healed tails. The association between disease conditions and tail lesions is difficult to assess in field studies, as data collection is laborious and most data are therefore routinely collected at herd level and at slaughterhouses. For example, Moinard et al. (41) found an association between respiratory disease, rectal prolapse, and TB. The association between diseases and TLs have been shown in studies using mostly pathological or slaughterhouse data. A connection has been found between TL and abscesses (7, 9, 26), arthritis or leg disorders (9, 26, 31), pericarditis (26), respiratory conditions (7, 9, 23, 26, 31), skin infections (26), and whole and partial carcass condemnations (26).

It is difficult to find results about the possible association between mortality and TLs. Moinard et al. (41) reported that a post-weaning mortality >2.5% was associated with a 3.9-fold increase in the risk of TB estimated on farms by researchers. Mortality is a very rough measure to estimate animal welfare and the decisions of the farmer when to cull an animal has a large effect on this figure. However, the association between higher annual mortality and increase in healed tails in our study can be considered to describe the long-term situation of welfare in the herd. The association of increased percentage of intact tails and decreased mortality also supports the same idea of mortality being associated with tail score in the slaughterhouse.

Our study further shows that it is possible to grow undocked pigs with only a small percentage of pigs with serious tail wounds at the slaughterhouse. In almost 20% of the herds (Figure 1), no pigs had major acute wounds upon arrival at the slaughterhouse. Further, when examining the percentage of intact tails, the best 10% of the herds had >79% fully intact tails and the very best herd had 93% fully intact tails. Further, according to the herd health visit data, in 51.2% of the herds, veterinarians had recorded > 95% of tails as intact. This figure is well in accordance with the result of our scoring at the slaughterhouse, when we included both intact and healed tails in the figure.

This study has some limitations. It was not possible to evaluate the same pigs in the herd and at the slaughterhouse. However, we believe that the risk factors and situation in the herds are quite constant except for occasional TB outbreaks. The fact that piggeries usually empty their finishing units in 3–4 deliveries may have caused selection of certain types of pigs in our slaughterhouse data of 1 week. For example, the last delivery group of the pigs in one room may include those that have grown slower than the ones that were sent to slaughterhouse in the first delivery group. Due to these two factors, we may not have identified all real associations between the risk factors in the herd and the tail scoring results at slaughterhouse. However, we believe that the ones that have been identified are correct. The herd visits were performed by several different veterinarians, which also caused variation in the evaluation. However, this variation is likely mitigated by common educational background (veterinary studies and Sikava course).

## Conclusions

Our results show that by recording only one type of tail condition such as tails with major acute wounds at the slaughterhouse, it is not possible to estimate the total tail lesion situation in herds before slaughter. Herds seem to have varying combinations of percentages of tails that were intact, healed, with minor acute wounds, and with major acute wounds. Each of these provide different information about the situation on the farm. Tail lesion scoring at the slaughterhouse appears to be a more precise measure than that performed on-farm as part of a herd health visit. Tail condition as measured at the slaughterhouse shows potential to estimate the long-term welfare level on the farm. This is related to management, such as the use of manipulable objects, and maybe also to some health parameters on the farm.

## Data Availability Statement

The data of the slaughtered pigs are owned by the producers. Those interested in the datasets should contact Anna Valros (anna.valros@helsinki.fi). The Board of Sikava gave the permit to use the data anonymously.

## Ethics Statement

Ethical review and approval was not required for the animal study because the data was collected firstly from database where veterinarians had stored their herd health evaluations and secondly in the slaughterhouse after stunning and bleeding. Written informed consent for participation was not obtained from the owners because the data from the database was anonymous.

## Author Contributions

AV was responsible for the data analysis. All authors contributed to the study design, data collection, interpretation of the results, preparation of the manuscript, participated in writing of the manuscript, and gave their approval for the final manuscript.

## Conflict of Interest

EV and JV are employees of the slaughterhouse company. The University of Helsinki was responsible for data analysis and scientific publication throughout the study. The company involvement did not have any effect on the study design, data collection, interpretation of results, or the writing of the manuscript. The remaining authors declare that the research was conducted in the absence of any commercial or financial relationships that could be construed as a potential conflict of interest.
